# Hypertension among Middle Eastern and North African adults residing in the United States: addressing equity in health research representation using the All of Us Research Program, 2000–2024

**DOI:** 10.3389/fmed.2026.1819597

**Published:** 2026-06-16

**Authors:** Eissa A. Jafari

**Affiliations:** Department of Pharmacy Practice, College of Pharmacy, Jazan University, Jazan, Saudi Arabia

**Keywords:** All of Us research Program, blood pressure, health research equity, hypertension, Middle Eastern and North African

## Abstract

**Background:**

Hypertension (HTN) is a leading modifiable risk factor for cardiovascular disease and mortality; however, its epidemiology among the Middle Eastern and North African (MENA) population residing in the United States (US) remains uncharacterized due to historical racial misclassification and underrepresentation in national datasets. This study aimed to evaluate HTN prevalence, treatment, control, and predictors among MENA adults using data from the All of Us Research Program (2000–2024).

**Methods:**

This was a retrospective cohort study of self-identified MENA adults aged ≥18 years with at least one outpatient blood pressure (BP) measurement. HTN was defined by an outpatient diagnosis of essential HTN. BP control was assessed using time in target range among participants with at least two outpatient BP measurements after index date and was defined as BP < 140/90 mm Hg ≥50% of outpatient visits. A stepwise logistic regression model was used to identify significant predictors of HTN.

**Results:**

Among 2,572 MENA adults included in the analysis, 18% (463) had HTN. Among hypertensive participants, 76% (352 of 463) received antihypertensive treatment, and 85% (400 of 463) achieved BP control. Participants aged ≤ 50 years demonstrated significantly higher BP control proportion, compared with those aged >50 years (97 vs. 83%, *p* = 0.0137). Significant predictors of HTN included diabetes, chronic kidney disease, coronary artery disease, hyperlipidemia, sleep apnea, obesity, male sex, age, anxiety, vitamin D deficiency, and anemia.

**Conclusions:**

Among US MENA adults that participated in the All of Us research Program, HTN prevalence was lower than estimates reported for the general US adult population, while treatment and BP control were higher. Cardiometabolic conditions were the primary drivers of HTN risk, with additional associations observed for vitamin D deficiency and anemia, highlighting the need for improved inclusion of MENA adults in national health research to inform targeted prevention and management strategies.

## 1 Introduction

Hypertension (HTN) remains the most prevalent modifiable risk factor for cardiovascularx, renal, and metabolic diseases worldwide, contributing to approximately 10.7 million deaths and 212 million disability-adjusted life years annually (1). Despite the availability of effective pharmacological therapies and increased public health initiatives, less than 20% of individuals with HTN globally achieve the recommended blood pressure (BP) target (2). In the United States (US), nearly half of all adults meet diagnostic criteria for HTN, yet a recent analysis indicated no significant improvements in HTN prevalence or control proportion in the post-pandemic era (3).

Significant disparities in HTN prevalence, awareness, treatment, control, and outcomes have been reported among US racial and ethnic groups. Non-Hispanic Black and Hispanic individuals experience higher HTN prevalence and lower proportion of awareness and BP control, compared with Non-Hispanic White individuals (4, 5). In contrast, data on HTN in Middle Eastern and North African (MENA) populations remain scarce, despite the rapid growth and increasing visibility of this group within the US (6). Approximately 3.7 million individuals of MENA descent reside in the US, constituting about 3% of the nation's immigrant population and encompassing considerable ethnic diversity, including Arabs, Iranians, Kurds, Afghans, and others (7). Notably, individuals from MENA backgrounds carry a unique cardiometabolic risk profile, influenced by high proportion of HTN, diabetes, and obesity in their countries of origin (8, 9). However, the MENA population remains largely invisible in US health research due to systematic racial misclassification. Federal databases and national surveys (e.g., NHANES, NHIS) do not recognize MENA as a distinct ethnic group, typically classifying them as White. This misclassification creates a “hidden minority” phenomenon that obscures epidemiologic patterns and limits the development of targeted, evidence-based public health strategies (6, 10, 11).

Evidence from a region-specific meta-analysis demonstrated that nearly one-quarter of adults in MENA countries are hypertensive; however, only 50% of affected individuals are aware of their diagnosis, and fewer than 20% have their BP controlled (12). In contrast, A US-based self-administered questionnaire study conducted on only 126 Arab Americans in Southern California reported a HTN prevalence of 37%, with awareness and control proportion of 67 and 46%, respectively (13). Nevertheless, this and similar other studies of MENA populations in the US have been limited by small sample sizes, non-representative sampling methods, and a lack of longitudinal or comprehensive data, limiting their generalizability to the broader MENA population (8, 14, 15). The absence of a standardized MENA identifier in national health records and surveys remains a major barrier to conducting research in this population (6, 10, 11).

The All of Us Research Program, launched by the National Institutes of Health, was established to overcome such challenges by recruiting over one million participants with deliberate oversampling of minority groups, including those of MENA ancestry. This nationwide resource provides secure, de-identified access to electronic health records (EHRs), survey data, physical measurements, and biospecimens, thereby offering an unparalleled opportunity to conduct research on HTN in this historically underrepresented population, which may not be feasible using traditional databases or local community recruitment (16, 17). Leveraging All of Us data, this study aimed to characterize the epidemiology of HTN in MENA adults residing in the US by assessing HTN prevalence, control, treatment patterns, and predictors. Findings from this study are intended to inform clinical practice, guide public health interventions, and support future research efforts aimed at reducing HTN-related disparities and improving cardiovascular health in MENA populations.

## 2 Method

### 2.1 Data source

This was a retrospective cohort study utilizing data from the All of Us Research Program (2000–2024), a large, prospective, nationwide initiative launched by the National Institutes of Health to accelerate health research and medical discovery. All of Us aims to recruit over one million individuals living in the US, with a strong emphasis on diversity and inclusion of historically underrepresented populations in biomedical research. The program collects a wide range of data types, such as EHRs, physical measurements, biospecimens, digital health data, and participant-reported survey responses (16, 17). For this study, we accessed the Controlled Tier Dataset (Version 8) through the All of Us Researcher Workbench, a secure, cloud-based platform. Data used in this study included EHRs (demographics, conditions, visits, prescriptions), participant-reported survey responses, and physical measurements. Because All of Us data are de-identified and collected under centralized Institutional Review Board (IRB) oversight, this study was deemed exempt from IRB approval under the program's public use guidelines.

### 2.2 Study population

We included adults aged ≥18 years, who self-identified as MENA and had at least one BP measurement recorded at an outpatient visit. Outpatient setting was selected because HTN diagnosis and BP measurements obtained in outpatient encounters are more representative of chronic BP status and consistent with guideline-based HTN assessment. The included cohort was divided into two groups: individuals with HTN and those without HTN. Participants with HTN were required to have at least one outpatient diagnosis for HTN using the Systemized Nomenclature of Medicine (SNOMED) diagnosis code (59621000). Participants classified as non-HTN were required to have no recorded diagnosis of HTN in their EHRs at any type of encounter. To ensure accurate classification, we excluded patients with a diagnosis of pregnancy.

The index date was defined as the first outpatient visit with a HTN diagnosis for hypertensive patients. For non-HTN individuals, the index date was defined as the first outpatient visit with a recorded BP measurement. This approach ensured consistent baseline characterization across groups.

### 2.3 Blood pressure control

BP control was evaluated among a subset of HTN patients with at least two outpatient BP measurements after the index date (400 of 463 patients), to ensure reliable estimation of BP control over time. We used the time in target range (TTR) method to define BP control. A total of 63 hypertensive participants were excluded from the BP control analysis due to insufficient BP measurements. Among included participants, the mean number of outpatient BP measurements was 27 visits. Consistent with the 2003 Seventh Joint National Committee (JNC7) guidelines, BP control was defined as BP values < 140/90 mm Hg (18). A sensitivity analysis using BP threshold of < 130/80 mm Hg was also performed. For each participant, TTR was calculated as the proportion of outpatient visits with BP values within the controlled range divided by the total number of outpatient visits (19). Participants with ≥50% of BP measurements within the target range were classified as having controlled BP, whereas those with < 50% of controlled BP measurements were classified as having uncontrolled BP.

### 2.4 Covariates

Demographic variables included age (< 50 years, ≥50 year), sex (male, female), and ethnicity (Hispanic or Latino, Not Hispanic or Latino). Socioeconomic variables included marital status (married, unmarried), employment status (employed, unemployed), education level (high school, college, advanced degree), insurance status (yes, no), income level (< 50K, 50–150K, >150), and birthplace (USA, other). We included lifestyle variables such as smoking status. Demographic, socioeconomic, and lifestyle variables were obtained from participant-reported survey responses within the All of Us Research Program. Body mass index (BMI; < 30 kg/m^2^, ≥30 kg/m^2^) was derived from the physical measurement data. A BMI of ≥30 kg/m^2^ was used to define obesity. Clinical comorbidities were captured at or before the index date to maintain temporal alignment when assessing factors associated with HTN and included anxiety, asthma, diabetes, hyperlipidemia, hyperthyroidism, vitamin D deficiency, and anemia. Comorbidities were identified using EHR-based condition records from the All of Us Research Program using standardized SNOMED diagnosis codes. Clinical comorbidities were identified using EHR-based SNOMED diagnosis records rather than self-reported survey responses because EHR diagnoses included diagnosis dates, allowing more reliable temporal alignment with the index date, and were less susceptible to recall and reporting bias. Most covariates had complete data. In cases of unavailable categorical data, such as income, participants were categorized as ‘unknown' and retained in the analysis.

Antihypertensive medications were identified from prescription records. Antihypertensive medications classes included angiotensin-converting enzyme inhibitors (ACEIs), angiotensin receptor blockers (ARBs), calcium channel blockers (CCBs), thiazide and thiazide-like diuretics, aldosterone antagonists, other diuretics (e.g., potassium-sparing diuretics, loop diuretics), beta-blockers (BBs), centrally acting alpha agonists, vasodilators, renin inhibitors, and alpha receptor blockers. Medication use was assessed from the index date through 12 months post-index date, consistent with HTN management guidelines that recommend an observation period for lifestyle modification and medication adjustment (18, 20).

### 2.5 Statistical analysis

Descriptive statistics were used to present demographics, socioeconomic, and clinical characteristics overall and across the HTN and non-HTN populations. Categorical variables were compared using Chi-square tests, and continuous variables using t-tests. Characteristics with cell counts below 20 were not reported in accordance with the All of Us Research Program Data and Statistics Dissemination Policy to ensure participant confidentiality

To identify significant factors associated with HTN, we first conducted univariate logistic regression analyses to evaluate associations between HTN and independent variables (Supplementary Table S1). Variables with a statistically significant association (*p* < 0.05) in univariate analyses were then included in a multivariable stepwise logistic regression model to identify independent predictors of HTN. The final multivariable logistic regression model was evaluated by assessing how well the model distinguished participants with and without hypertension using the c-statistic and assessing the overall model fit using the Hosmer-Lemeshow goodness-of-fit test. Adjusted odds ratios (ORs) and 95% confidence intervals (CIs) were reported. All analyses were conducted using SAS version 9.4 (SAS Institute Inc., Cary, NC) and R version 4.3.3 (R Foundation for Statistical Computing, Vienna, Austria). A two-sided *p*-value < 0.05 was considered statistically significant.

## 3 Results

### 3.1 Cohort selection and patient characteristics

**Figure 1:**
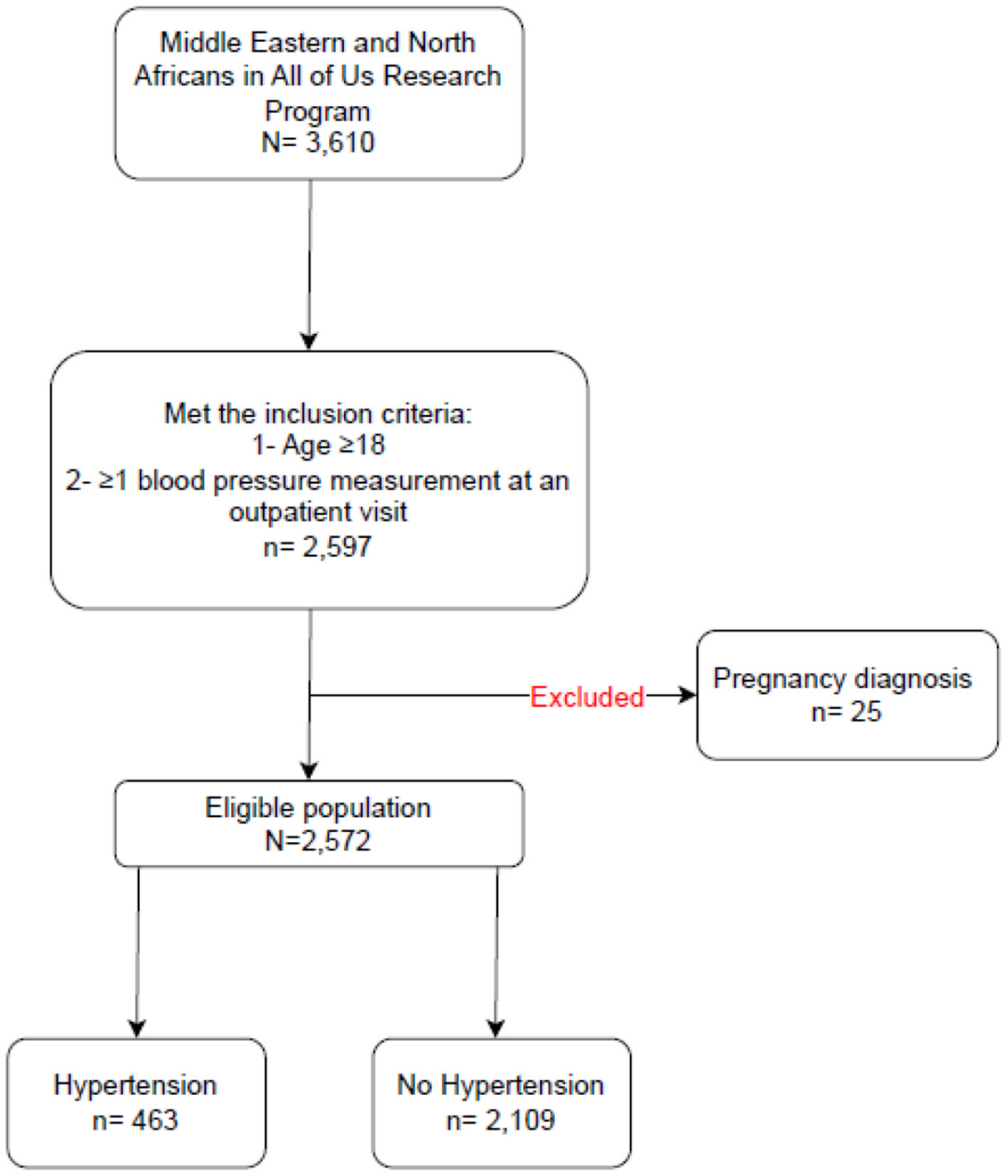
Flowchart of data preparation and cohort selection.

A total of 3,610 MENA adults were initially identified from the All of Us Research Program. After applying the inclusion criteria of age ≥18 years and the availability of at least one BP measurement recorded during an outpatient visit, 2,597 individuals were eligible for screening. Twenty-five participants with a pregnancy diagnosis were excluded, resulting in a final cohort of 2,572 participants. Of these, 463 (18%) had a diagnosis of HTN, while 2,109 (82%) did not (Figure 1).

Patients' characteristics at the index date overall and by HTN status are presented in Table 1. The mean age of the cohort was 49 years, and 47% were male. Participants with HTN were significantly older than those without HTN (66 vs. 46 years, *p* < 0.0001), with 85% of hypertensive participants aged >50 years compared with 63% in the non-hypertensive group (*p* < 0.0001). There were significantly more males among hypertensive participants (61 vs. 44%, *p* < 0.0001), compared with non-hypertensive participants. In contrast, Hispanic ethnicity was significantly higher in non-hypertensive participants (6 vs. 4%, *p* = 0.0354), compared with hypertensive patients.

**Table 1:** Characteristics of the MENA study population at the index date overall and by HTN status.

Characteristics^*^	Overall (*N* = 2,572)	HTN (*n* = 463)	No HTN (*n* = 2,109)	*p*-value
Demographic				
Age mean std	49 (18)	66 (14)	46 (16)	< 0.0001
Age category				< 0.0001
≤ 50 years	1,166(45%)	68 (15%)	771 (37%)	
>50 years	1,406 (55%)	395 (85%)	1,338 (63%)	
Sex at birth				< 0.0001
Male	1,210 (47%)	281 (61%)	929 (44%)	
Female	1,340 (52%)	179 (39%)	1,161 (56%)	
Ethnicity				0.0354
Hispanic or Latino	151 (6%)	18 (4%)	133 (6%)	
Not Hispanic or Latino	2,421 (94%)	445 (96%)	1,976 (94%)	
Socioeconomic			
Education				0.6079
High school	278 (11%)	55 (12%)	223 (11%)	
College	1,275 (50%)	236 (51%)	1,039 (49%)	
Advanced degree	972 (38%)	165 (36%)	807 (38%)	
Employment				< 0.0001
Employed	1,545 (60%)	204 (44%)	1,341 (64%)	
Unemployed	938 (36%)	241 (52%)	697 (33%)	
Income				0.0392
< 50k	420 (16%)	66 (14%)	354 (17%)	
50–150k	660 (26%)	106 (22%)	554 (26%)	
>150k	681 (26%)	120 (26%)	561 (27%)	
Unknown	811 (32%)	171 (36%)	640 (30%)	
Insurance				0.0092
Yes	2,395 (93%)	445 (96%)	1,950 (92%)	
No	91 (4%)	8 (2%)	83 (4%)	
Marital status				< 0.0001
Married	1,290 (50%)	279 (60%)	1,011 (48%)	
Unmarried	1,232 (48%)	178 (38%)	1,054 (50%)	
Birthplace				0.0005
USA	993 (39%)	146 (32%)	847 (40%)	
Other	1,579 (61%)	317 (68%)	1,262 (60%)	
BMI				< 0.0001
BMI < 30 kg/m^2^	1,881 (73%)	260 (56%)	1,621 (77%)	
BMI ≥30 kg/m^2^	691 (27%)	203 (44%)	488 (23%)	
Family history of HTN				0.0627
Yes	693 (27%)	141 (30%)	552 (26%)	
No	1,879 (73%)	322 (70%)	1,557 (74%)	
Lifestyle				
Smoking				0.0664
No	1,173 (46%)	229 (49%)	944 (45%)	
Comorbidities				< 0.0001
Anxiety	178 (7%)	72 (16%)	106 (5%)	
Asthma	97 (4%)	43 (9%)	54 (3%)	< 0.0001
Diabetes	160 (6%)	128 (28%)	32 (2%)	< 0.0001
Hyperlipidemia	350 (14%)	233 (50%)	117 (6%)	< 0.0001
Hypothyroidism	104 (4%)	48 (10%)	56 (3%)	< 0.0001
Vitamin D deficiency	119 (5%)	57 (12%)	62 (3%)	< 0.0001
Anemia	144 (6%)	75 (16%)	69 (3%)	< 0.0001

^*^Characteristics with cell counts below 20 were not reported in accordance with the All of Us Research Program Data and Statistics Dissemination Policy to ensure participant confidentiality.

These characteristics include alcohol consumption, delay of care, atherosclerosis, coronary artery disease, cardiomegaly, chronic kidney disease, chronic obstructive pulmonary disease, gastroesophageal reflex disease, heart failure, sleep apnea, stroke, myocardial infarction, and Alzheimer's disease and related dementia.

MENA, Middle Eastern and North Africa; HTN, hypertension; USA, United States of America; BMI, body mass index.

There was no significant difference in the education between the two groups. However, compared with non-hypertensive participants, those with HTN were less often employed (44 vs. 64%, *p* < 0.0001), more likely to be married (60 vs. 48%, *p* < 0.0001), and more often born outside the US (68 vs. 60%, *p* = 0.0005). Hypertensive participants also reported insurance coverage significantly more often (96 vs. 92%, *p* = 0.0092), compared to those without HTN (Table 1).

Obesity (BMI ≥30 kg/m^2^) was significantly more common among participants with HTN, compared to those without HTN (44 vs. 23%, *p* < 0.0001). Smoking and family history of HTN did not differ significantly between the two groups. In contrast, compared with non-hypertensive participants, those with HTN reported comorbidities significantly more often, including hyperlipidemia (50 vs. 6%), diabetes (28 vs. 2%), anemia (16 vs. 3%), anxiety (16 vs. 5%), vitamin D deficiency (12 vs. 3%), hypothyroidism (10 vs. 3%), and asthma (9 vs. 3%; all *p* < 0.0001; Table 1).

### 3.2 Antihypertensive prescriptions

**Figure 2:**
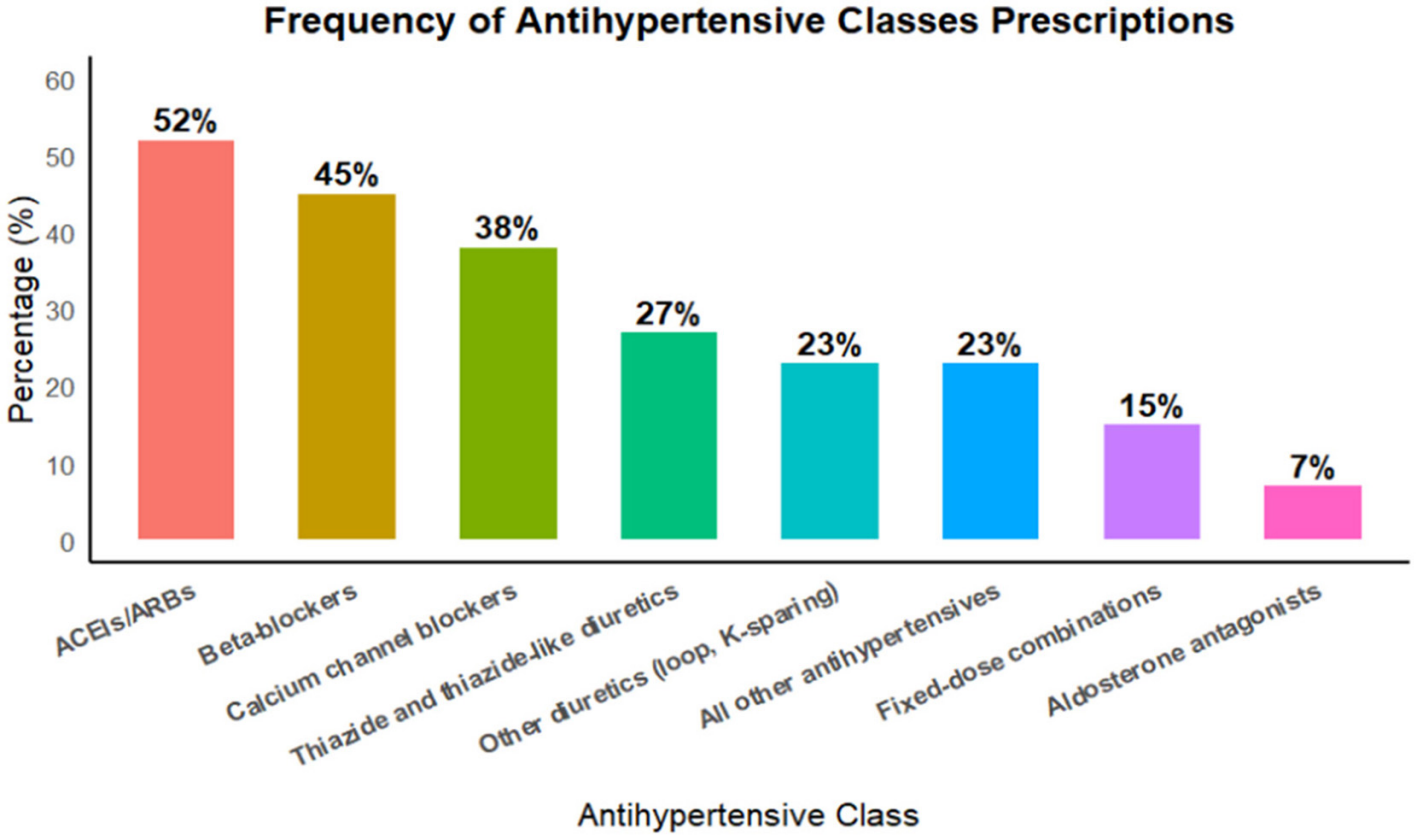
Frequency of antihypertensive classes in the MENA study population (*n* = 352). ACEIs, angiotensin-converting enzyme inhibitors; ARBs, angiotensin receptor blockers.

Among hypertensive participants, 76% (352) were prescribed antihypertensive therapy. The most commonly prescribed drug classes were ACEIs or ARBs (52%), followed by BBs (45%), CCBs (38%), and thiazide or thiazide-like diuretics (27%). Other diuretics and other antihypertensive agents were each prescribed to 23% of patients, while fixed-dose combination therapies and aldosterone antagonists were prescribed to 15 and 7% of participants, respectively (Figure 2).

### 3.3 Blood pressure control

**Figure 3:**
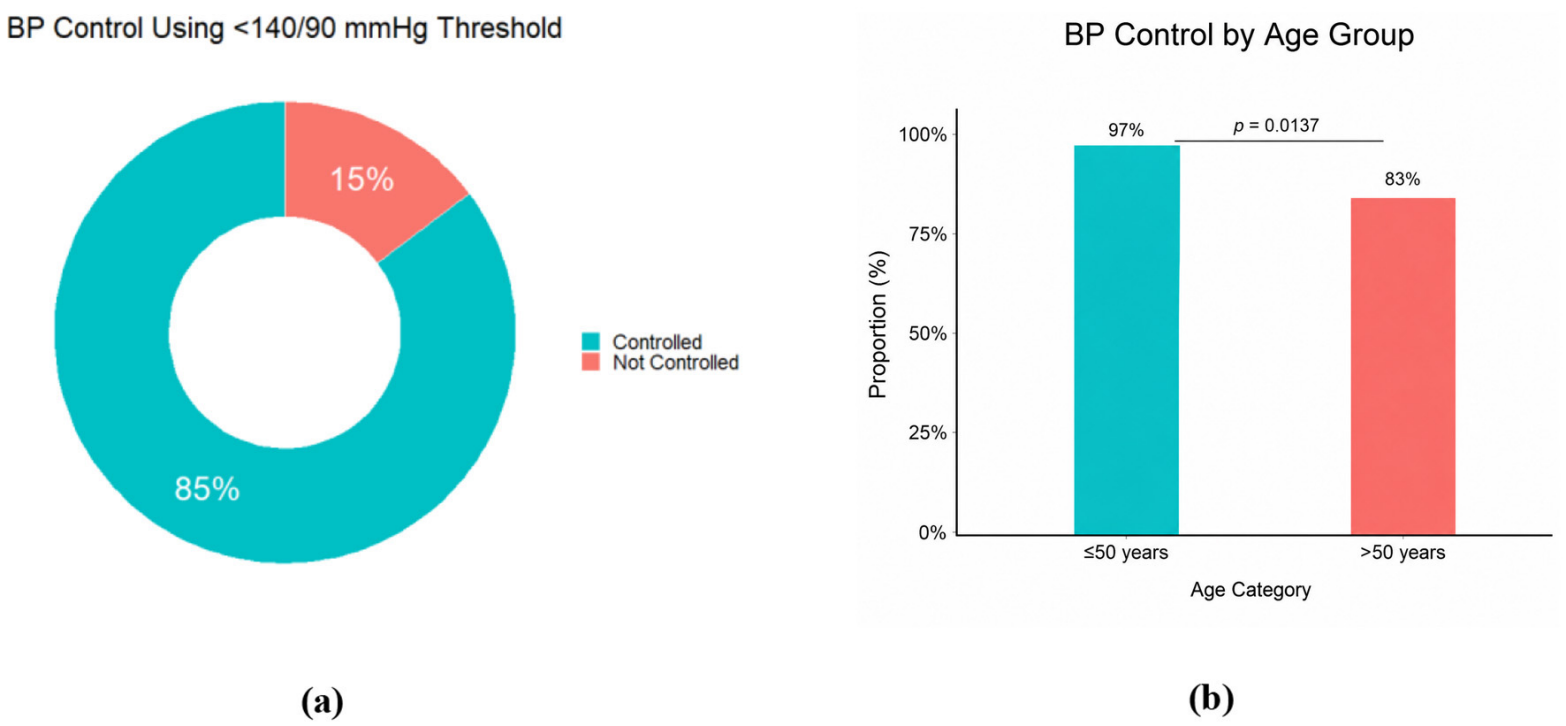
BP control among hypertensive MENA adults (*n* = 400): **(a)** overall BP control in the hypertensive cohort; **(b)** BP control stratified by age. BP, blood pressure; MENA, Middle Eastern and North African.

Overall, 85% (341 of 400) of participants with HTN who had at least two outpatient BP measurements achieved BP control (< 140/90 mm Hg; Figure 3a). Participants aged ≤ 50 years demonstrated significantly higher BP control (< 140/90 mm Hg) proportion compared with those aged >50 years (97 vs. 83%, *p* = 0.0137; Figure 3b). A sensitivity analysis using BP cut-off of < 130/80 mm Hg showed BP control of 40% (162; Supplementary Figure S1).

### 3.4 Predictors of hypertension in MENA

Significant predictors of HTN in the MENA study population are presented in Table 2. The final model showed good ability to distinguish participants with and without HTN (c-statistic = 0.90). No evidence of problematic multicollinearity was identified, with all variance inflation factor values below 1.5. The Hosmer-Lemeshow test indicated acceptable overall model fit (*p* = 0.5601). The predictors included diabetes (OR = 8.44; 95% CI: 5.13–13.87, *p* < 0.0001), chronic kidney disease (CKD; OR = 7.03; 95% CI: 2.46–20.11, *p* = 0.0003), coronary artery disease (CAD; OR = 4.75; 95% CI: 2.19–10.33, *p* < 0.0001), hyperlipidemia (OR = 3.76; 95% CI: 2.68–5.28, *p* < 0.0001), sleep apnea (OR = 3.62; 95% CI: 1.74–7.50, *p* = 0.0006), anemia (OR = 2.97; 95% CI: 1.79–4.94, *p* < 0.0001), anxiety (OR = 2.76; 95% CI: 1.75–4.37, *p* < 0.0001), vitamin D deficiency (OR = 2.06; 95% CI: 1.19–3.57, *p* = 0.0103), male sex (OR = 1.75; 95% CI: 1.32–2.33, *p* = 0.0004), obesity (OR = 1.60; 95% CI: 1.19–2.14, *p* = 0.0018), and age (OR = 1.06; 95% CI: 1.05–1.07, *p* < 0.0001).

**Table 2:** Significant predictors of HTN in the MENA study population (*N* = 2,572).

Independent predictor	Odds ratio	Lower 95% CI	Upper 95% CI	*p*-value
Age	1.06	1.05	1.07	< 0.0001
Male vs. female	1.75	1.32	2.33	0.0004
Obesity (yes vs. no)	1.60	1.19	2.14	0.0018
Anxiety (yes vs. no)	2.76	1.75	4.37	< 0.0001
CAD (yes vs. no)	4.75	2.19	10.33	< 0.0001
CKD (yes vs. no)	7.03	2.46	20.11	0.0003
Diabetes (yes vs. no)	8.44	5.13	13.87	< 0.0001
Hyperlipidemia (yes vs. no)	3.76	2.68	5.28	< 0.0001
Sleep Apnea (yes vs. no)	3.62	1.74	7.50	0.0006
Vitamin D deficiency (yes vs. no)	2.06	1.19	3.57	0.0103
Anemia (yes vs. no)	2.97	1.79	4.94	< 0.0001

MENA, Middle Eastern and North Africa; HTN, hypertension; CAD, coronary artery disease; CKD, chronic kidney disease; CI, confidence interval.

## 4 Discussion

HTN remains a leading modifiable risk factor for cardiovascular disease globally, yet evidence on its epidemiology in the MENA population remains limited. Historically, MENA individuals have been underrepresented in health research and often misclassified as White in administrative and surveillance data, limiting the ability to assess their cardiometabolic burden and develop tailored interventions. To address this gap, this study aimed to examine HTN prevalence, treatment, control, and predictors among MENA adults using data from the All of Us Research Program. HTN prevalence was 18%; among those with HTN, 76% were prescribed antihypertensive medications, and 85% achieved BP control (< 140/90 mm Hg). BP control was significantly higher in younger patients, compared with older patients. Additionally, diabetes, CKD, CAD, sleep apnea, hyperlipidemia, vitamin D deficiency, anemia, obesity, and anxiety emerged as significant predictors of HTN in the MENA study population. To our knowledge, this study represents one of the largest US-based analyses of HTN epidemiology in MENA adults using the All of Us Research Program, overcoming limitations of prior studies.

The observed HTN prevalence of 18% among MENA adults in outpatient settings was lower than the contemporary estimates for the general US adult population, where recent national data reported a HTN prevalence of 48% among adults aged ≥18 (3). Comparative data from other races showed that non-Hispanic Black adults have the highest prevalence of HTN in the US (57%), followed by non-Hispanic Whites (44%), and Hispanics (44%), highlighting substantial racial and ethnic disparities (21). Beyond the US population, meta-analyses from the MENA region reported a HTN prevalence of 26%, slightly higher than the prevalence observed in the present cohort (12, 22). Similarly, in a US-based study of a community sample of Arab Americans in Southern California, the HTN prevalence was 37% (13). However, these comparisons should be interpreted cautiously because the present study was restricted to participants engaged in outpatient care and relied on outpatient HTN diagnoses to identify HTN patients, whereas prior US and MENA estimates were derived from population-based, community-based, or nationally representative cohorts with different healthcare engagement and HTN ascertainment methods. Additionally, the MENA cohort in this study had a relatively younger age distribution (mean age 49 years) than the general US population, which may have contributed to the lower observed HTN prevalence.

Treatment and control proportions in this MENA cohort were higher than those observed in US national and MENA study population data, with treatment and control proportion of 76 and 85%, respectively. In the US, 68% of adults with HTN are prescribed antihypertensive medications, with a control proportion (defined as BP < 140/90 mm Hg) of 48% (23). Across major racial and ethnic groups, treatment and control proportions vary significantly, with non-Hispanic Black adults demonstrating proportions of 67 and 39%, Hispanic adults 61 and 40%, and non-Hispanic White adults 67 and 49%, respectively (4). Compared to the MENA region, treatment and control proportions remain higher in this US cohort. Pooled estimates from MENA countries reported that only 41% of hypertensive adults received antihypertensive treatment, and 19% achieved BP control (12). Prior US-based study of MENA adults has likewise reported lower treatment and control proportion (52 and 46%, respectively) (13). These differences should be interpreted in the context of cohort characteristics and study design, as individuals engaged in outpatient care may be more likely to receive antihypertensive treatment and achieve BP control than general or community-based populations. Because most BP measurements in this cohort were obtained before implementation of the 2017 ACC/AHA guideline, BP control was evaluated using < 140/90 mm Hg threshold to facilitate comparability with prior US and MENA population studies. However, in the sensitivity analysis using the more stringent (< 130/80 mm Hg) threshold, BP control proportions were substantially lower, demonstrating the impact of BP threshold selection on reported HTN control estimates.

Several factors likely explain the relatively lower prevalence and higher treatment and control proportion observed among MENA adults in this study. First, participants in the All of Us cohort demonstrated a favorable socioeconomic profile, with nearly 90% reporting a college or advanced degree, and over 90% having health insurance coverage. Higher educational attainment and insurance access are strongly associated with increased health literacy, improved healthcare access, regular monitoring, and greater engagement in preventive care, all of which contribute to lower HTN burden and better management and control once diagnosed (24, 25). Second, the All of Us recruitment framework may have introduced a “healthy volunteer effect,” as participants are often more health-conscious, more engaged with healthcare systems, and more likely to participate in preventive care than the general population. This may have contributed to underestimation of HTN prevalence and overestimation of antihypertensive treatment and BP control proportions in this cohort. Third, methodological factors likely contributed to the observed findings. The inclusion of participants actively engaged in outpatient care, combined with the requirement for documented BP measurements, likely facilitated more consistent HTN monitoring and management, contributing to the high BP control proportion. Additionally, exclusion of pregnancy, use of validated EHR diagnoses, and reliance on outpatient BP documentation may have reduced measurement error and recall bias, which frequently inflate prevalence estimates in other studies. Fourth, higher treatment and control proportions may reflect favorable aspects of the US healthcare system, including widespread availability of antihypertensive medications, integrated chronic disease management, and adherence to standardized HTN treatment guidelines, compared with limited access and fragmented healthcare systems in many MENA countries. Finally, cultural and structural factors may also play a role. MENA immigrants may arrive with lower baseline cardiometabolic risk, with risk accumulating over time through acculturation. Those who remain engaged in healthcare systems may be more likely to receive and adhere to treatment, contributing to the higher BP control proportion observed here (26).

Consistent with prior literature, the strongest predictors of HTN in this MENA cohort clustered around cardiometabolic conditions, including diabetes, CKD, CAD, hyperlipidemia, and obesity. These associations align with extensive evidence from both US and MENA-region studies, reinforcing the central role of metabolic syndrome components in driving HTN risk (1, 22, 27). Sleep apnea also emerged as a significant factor, consistent with growing recognition of its role in HTN pathophysiology through mechanisms involving nocturnal hypoxia, sympathetic activation, and vascular dysfunction (28). Additionally, the observed association between anxiety and HTN further supports the emerging evidence linking psychosocial stress and autonomic dysregulation to elevated BP, although this relationship remains less well characterized in the MENA population (29).

Interestingly, vitamin D deficiency and anemia were independent predictors of HTN in this study. While less frequently reported in US HTN literature, these findings are biologically plausible and consistent with their high prevalence in MENA populations (30, 31). Vitamin D deficiency has been implicated in endothelial dysfunction and dysregulation of the renin–angiotensin–aldosterone system, mechanisms that increase vascular tone and BP (32). Anemia, often reflecting nutritional or inflammatory states, may contribute to HTN through compensatory increases in cardiac output, oxidative stress, and chronic vascular remodeling (33). These findings suggest that, although the core cardiometabolic drivers of HTN in MENA adults resemble those observed in other US racial and ethnic groups, additional factors such as vitamin D deficiency and anemia may represent population-specific risk pathways. Integrating nutritional, metabolic, and psychosocial domains into HTN risk assessment may enhance HTN prevention and management strategies in this population.

Our study is not without limitations. First, while the retrospective cohort design allowed temporal alignment between predictors and HTN onset, causal inference remains limited, as residual confounding from unmeasured or unobserved variables cannot be excluded. Second, selection bias may limit generalizability, as All of Us participants tend to be more health-conscious, digitally literate, and engaged in preventive care than the broader MENA population. Third, EHR-based BP measurements may vary across clinical settings in terms of frequency, technique, and documentation, potentially leading to misclassification of HTN status or BP control. Restricting comorbidities to EHR-based diagnoses recorded at or before the HTN index date, may have underestimated the prevalence of some comorbidities among MENA adults receiving outpatient care. Prescription records capture medication orders but not adherence, which might have influenced the antihypertensive utilization estimates. Fourth, BP control was defined using the 140/90 mm Hg threshold; however, most of the BP measurements in this study pre-dated the adoption of lower guideline-recommended targets (18, 34). The status of HTN was determined by a HTN diagnosis at outpatient visits, which might explain the lower HTN prevalence; however, we wanted to identify stable and clinically recognized HTN cases. Additionally, some participants may not have had multiple outpatient BP measurements or antihypertensive medication records documented on the same date as the HTN diagnosis in the EHR, which limited the feasibility of incorporating these criteria into the baseline HTN definition and may have resulted in exclusion of otherwise clinically recognized HTN cases. Lastly, some variables relied on self-reported data (e.g., smoking and alcohol use), which are susceptible to recall and social desirability bias that may vary by cultural background or language proficiency. Because All of Us surveys were primarily administered in English and Spanish, language barriers may have influenced participation, self-identification, and the accuracy of self-reported responses among some MENA participants with limited English or Spanish proficiency. Despite these limitations, our study is the first to analyze HTN epidemiology in a large, self-identified MENA cohort residing in the US, addressing the longstanding research gap created by misclassification of this group in national data systems. Leveraging the All of Us dataset enabled integration of clinical, survey, and demographic data, allowing a multidimensional characterization of HTN prevalence, treatment, control, and predictors. The availability of repeated BP measurements further strengthened the assessment of BP control over time, reducing reliance on single-visit measurements that often overestimate HTN prevalence and control.

## 5 Conclusion

This study provided a large population-level characterization of HTN prevalence, treatment, control, and predictors among MENA adults in outpatient settings using data from the All of Us Research Program. The study showed a comparatively lower HTN prevalence but higher treatment and BP control proportions relative to national estimates and prior MENA-focused studies. Cardio metabolic conditions emerged as strong predictors of HTN, while novel associations with vitamin D deficiency and anemia offer new insight into potential biological and population-specific determinants of HTN risk. By addressing a major gap resulting from the historical misclassification of MENA individuals in the US and their underrepresentation in health research, this study establishes foundational evidence for HTN epidemiology in this population. However, because the All of Us cohort may over represent individuals with higher educational attainment, insurance coverage, and healthcare engagement, the findings may not be generalizable to all US MENA adults. Our findings highlight the importance of continued inclusion of MENA populations in national datasets and the development of culturally informed strategies to reduce HTN disparities and promote cardiovascular health equity in the US.

## Data Availability

The datasets presented in this article are not readily available because this study used data from the All of Us Research Program's Controlled Tier Dataset version 8, available to authorized users on the Researcher Workbench at https://workbench.researchallofus.org/login. Requests to access the datasets should be directed to https://workbench.researchallofus.org/login.
