# Supplementary material for: Hypertension among Middle Eastern and North African adults residing in the United States: addressing equity in health research representation using the All of Us Research Program, 2000–2024
Source: Front Med (Lausanne). 2026 Jun 16;13:1819597. doi: 10.3389/fmed.2026.1819597 (PMC13314769; doi:10.3389/fmed.2026.1819597)
Supplement: Supplementary file 1 [file Supplementary_file_1.docx]

**Supplementary Materials**

| **Table S1. Results of univariate logistic regression** | | | | |
| --- | --- | --- | --- | --- |
| variables | Odds ratio | Lower 95% CI | Upper 95% CI | P-Value |
| Age | 1.075 | 1.067 | 1.083 | <.0001 |
| Male vs Female | 1.962 | 1.596 | 2.41 | 0.0389 |
| Hispanic vs Not Hispanic | 0.601 | 0.363 | 0.994 | 0.0472 |
| Smoker Yes vs No | 0.828 | 0.677 | 1.013 | 0.0662 |
| College vs Advanced Degree | 1.111 | 0.892 | 1.383 | 0.5664 |
| High School vs Advanced Degree | 1.206 | 0.859 | 1.69 | 0.3228 |
| Unemployed vs Employed | 2.273 | 1.847 | 2.798 | 0.0003 |
| 50-100k vs More than 100k | 0.895 | 0.672 | 1.191 | 0.2564 |
| Less than 50k vs More than 100k | 0.872 | 0.628 | 1.211 | 0.2307 |
| Insurance No vs Yes | 0.422 | 0.203 | 0.879 | 0.1500 |
| Unmarried vs Married | 0.612 | 0.498 | 0.753 | 0.5547 |
| Birthplace Not USA vs USA | 1.457 | 1.176 | 1.806 | 0.0006 |
| Obese Yes vs No | 2.594 | 2.103 | 3.198 | <.0001 |
| Asthma 1 vs 0 | 3.896 | 2.575 | 5.895 | <.0001 |
| Anxiety 1 vs 0 | 3.480 | 2.531 | 4.784 | <.0001 |
| Atherosclerosis 1 vs 0 | 23.619 | 13.356 | 41.766 | <.0001 |
| CAD 1 vs 0 | 35.699 | 18.766 | 67.909 | <.0001 |
| Cardiomegaly 1 vs 0 | 14.068 | 5.089 | 38.892 | <.0001 |
| CKD 1 vs 0 | 23.044 | 10.128 | 52.435 | <.0001 |
| COPD 1 vs 0 | 35.019 | 10.436 | 117.507 | <.0001 |
| diabetes 1 vs 0 | 24.800 | 16.554 | 37.152 | <.0001 |
| GERD 1 vs 0 | 6.719 | 4.898 | 9.218 | <.0001 |
| Heart failure 1 vs 0 | 12.537 | 4.879 | 32.212 | <.0001 |
| Hyperlipidemia 1 vs 0 | 17.247 | 13.289 | 22.383 | <.0001 |
| Sleep apnea 1 vs 0 | 15.837 | 8.931 | 28.081 | <.0001 |
| Vitamin D Deficiency 1 vs 0 | 4.635 | 3.186 | 6.745 | <.0001 |
| Anemia 1 vs 0 | 5.715 | 4.049 | 8.065 | <.0001 |
| Hypothyroidism 1 vs 0 | 4.240 | 2.843 | 6.324 | <.0001 |
| Abbreviations: CAD: coronary artery disease, CKD: chronic kidney disease, COPD: chromic obstructive pulmonary disease, GERD: gastroesophageal reflux disease | | | | |


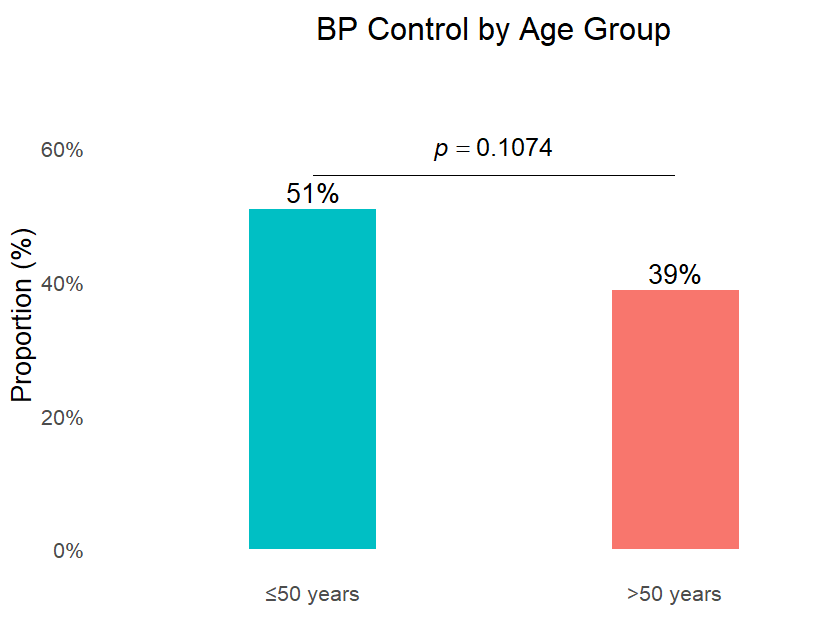

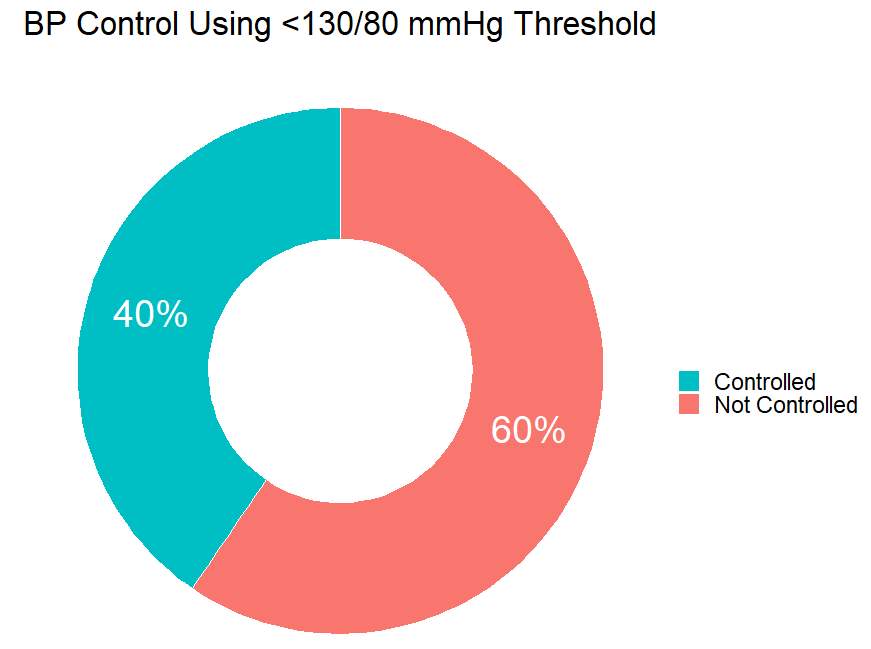


**(b)**

**(a)**

**Figure S1.** **BP control among hypertensive MENA adults using cut-off of 130/80 mm Hg (n=400).** (a) overall BP control in the hypertensive cohort; (b) BP control stratified by age. Abbreviations: BP: blood pressure, MENA: Middle Eastern and North African
